# Engineering Polymorphic Phase Boundary in Aerosol-Deposited Ba(Zr*_x_*Ti_1−_*_x_*)O_3_ Thick Films for Large Transverse Piezoelectricity

**DOI:** 10.3390/nano16060352

**Published:** 2026-03-13

**Authors:** Jinlin Yang, Long Teng, Zhenwei Shen, Wenjia Zhang, Shuping Li, Hanfei Zhu, Hongbo Cheng, Yongguang Xiao

**Affiliations:** 1 Key Laboratory of Low Dimensional Materials and Application Technology of Ministry of Education, School of Materials Science and Engineering, Xiangtan University, Xiangtan 411105, China; 2 Institute of Advanced Energy Materials and Chemistry, School of Chemistry and Chemical Engineering, Qilu University of Technology, Jinan 250353, China; 3 Hunan Provincial Key Laboratory of Thin Film Materials and Devices, School of Materials Science and Engineering, Xiangtan University, Xiangtan 411105, China

**Keywords:** aerosol deposition, Ba(Zr*_x_*Ti_1−_*_x_*)O_3_ thick films, transverse piezoelectric coefficient |*e*_31,_ *_f_*|, phase coexistence

## Abstract

Conventional deposition techniques hinder the integration of high-performance lead-free piezoelectric thick films on silicon substrates due to slow growth kinetics and complex processing. Herein, dense, crack–free Ba(Zr*_x_*Ti_1−_*_x_*)O_3_ (BZT, *x* = 0–0.10) thick films (~2 μm) were fabricated via aerosol deposition (AD) followed by annealing, forming a nanocrystalline microstructure with an average grain size of ~78 nm. Compositional tuning showed optimal electromechanical performance at *x* = 0.03, attributed to the coexistence of tetragonal and orthorhombic phases near room temperature that reduce the phase transformation energy barrier. The optimized BZT films exhibit excellent electrical properties: saturation polarization of 31.3 μC/cm^2^, relative permittivity of 430, dielectric tunability figure of merit (FOM) of 155, and a large transverse piezoelectric coefficient |e_31,_ *_f_*| of 1.01 C/m^2^—comparable to textured magnetron–sputtered BaTiO_3_ films but with higher deposition efficiency. This work provides a high-throughput route for fabricating piezoelectric thick films, highlighting the potential of compositionally engineered AD–processed BZT in lead-free MEMS applications.

## 1. Introduction

Piezoelectric materials constitute the operational basis for a diverse spectrum of micro-electromechanical systems (MEMSs), ranging from precision actuators and acoustic sensors to vibrational energy harvesters [1,2,3]. For decades, lead-based compositions, particularly lead zirconate titanate (PZT), have dominated the field owing to their exceptional electromechanical properties [4,5]. However, the intrinsic toxicity of lead has raised significant environmental and health concerns, leading to stringent global regulations such as the Restriction of Hazardous Substances (RoHS) directive. Consequently, the development of high–performance, lead-free piezoelectric alternatives becomes a critical and urgent imperative for the sustainable future of electronic devices. Among the family of lead–free candidates, BaTiO_3_ (BTO)–based perovskites have emerged as one of the most promising systems due to their strong ferroelectricity and notable piezoelectric response [6,7,8]. However, translating these enhanced properties into a thin or thick film format compatible with silicon–based MEMS integration remains a formidable challenge.

Various deposition techniques, including sol–gel, metal–organic chemical vapor deposition (MOCVD), pulse laser deposition (PLD) and magnetron sputtering, have been employed to fabricate piezoelectric films on silicon [9,10,11,12]. However, a principal obstacle in translating the potential of advanced piezoelectric materials into practical MEMS devices is the requirement for ‘thick films’—layers on the micrometer scale necessary for generating the substantial force, displacement, or power demanded by actuators, sensors and energy harvesters [13,14]. Conventional thin-film techniques (e.g., sol–gel, magnetron sputtering) suffer from slow growth kinetics, making the fabrication of micrometer-thick layers prohibitively time-consuming and expensive in setup or raw materials [15]. Conversely, traditional bulk ceramic processing involves high sintering temperatures (>1300 °C) that are incompatible with silicon-based MEMSs, difficult to fabricate micrometer-level pellets with, and susceptible to cracking during packaging and operation [16]. This dilemma highlights the urgent need for an alternative synthesis route: a rapid, low-thermal-budget process capable of depositing dense, high-quality thick films directly onto substrates in a single, efficient step, which is essential to harness the performance of BZT for next–generation MEMSs.

To address the film growth kinetic limitations of conventional methods, Aerosol Deposition (AD) presents a compelling yet challenging alternative for fabricating thick films. Based on the mechanism of room temperature impact consolidation (RTIC), AD enables the rapid formation of highly dense, crack–free ceramic thick films at deposition rates several orders of magnitude higher than sputtering, offering a clear advantage for integrating thick piezoelectric layers on silicon [17]. On the other hand, the energetic particle bombardment inherent to the AD process also introduces high residual stresses, interface destruction and grain refinement (often down to the nanoscale) [18], which can severely suppress the ferroelectric response of the as–deposited films. While post–deposition annealing is commonly employed to recover crystallinity, pure BT thick films often exhibit limited piezoelectricity and temperature stability compared to their lead–based counterparts [19,20,21,22]. Merely recovering the grain structure is insufficient; to achieve a significant rise in electromechanical performance, the thermodynamic landscape of the material must be engineered.

Inspired by the successful design principle of Pb(Zr*_x_*Ti_1−_*_x_*)O_3_ (PZT), compositional engineering to create a morphotropic phase boundary (MPB) or a polymorphic phase boundary (PPB) with phase coexistence has been a key strategy to enhance their performance [23,24]. Within the Ba(Zr*_x_*Ti_1−_*_x_*)O_3_ (BZT) system, the substitution of Zr^4+^ for Ti^4+^ is known to induce a convergence of polymorphs, typically tetragonal (T) and orthorhombic (O) phases, in the vicinity of room temperature. Thermodynamically, this phase coexistence creates a flattened free energy profile, thereby significantly reducing the energy barriers associated with field-induced phase transformation and polarization rotation [25,26]. Despite extensive studies on bulk BZT, the interplay between Zr–doping, phase evolution, and electromechanical performance within the unique microstructural regime of AD thick films remains largely unexplored.

Herein, we report the fabrication of dense, crack–free BZT thick films (~2 μm) on silicon substrates using the AD method. By rigorously mapping the Zr–doping effects, we demonstrate that the electromechanical performance is maximized at a critical composition of *x* = 0.03. This enhancement is attributed to the engineered coexistence of tetragonal and orthorhombic phases, which facilitates field-induced phase transformation and polarization rotation. The optimized thick films deliver superior electrical properties, including a saturation polarization of 31.3 μC/cm^2^, a relative permittivity of 430, and most notably, a large transverse piezoelectric coefficient |*e*_31,_ *_f_*| of 1.01 C/m^2^. This piezoelectric response is comparable to that of sputtered textured BaTiO_3_ films [27], but is achieved with orders–of–magnitude higher deposition rates. This study provides a commercially viable and efficient pathway for fabricating high-performance piezoelectric thick films, highlighting the immense potential of compositionally tuned, AD–deposited BZT for next–generation lead–free MEMS applications.

## 2. Materials and Methods

### 2.1. Synthesis of Ba(Zr_x_Ti_1−x_)O_3_ Powders

A series of Ba(Zr*_x_*Ti_1−_*_x_*)O_3_ (BZT) powders, with compositions spanning (*x* = 0 to 0.10), were synthesized via a conventional solid-state reaction route. High-purity raw materials—BaCO_3_, ZrO_2_, and TiO_2_ (all 99% purity)—were weighed according to stoichiometric ratios and ball-milled in ethanol for 20 h. The dried mixtures underwent calcination at 850 °C for 1 h, followed by sintering at 1300 °C for 5 h to complete the perovskite phase formation. The resulting sintered ceramics powders were then crushed and ball-milled again for 10 h to obtain fine, sub–micron powders suitable for the aerosol deposition process.

### 2.2. Fabrication of BZT Thick Films via Aerosol Deposition

**Figure 1:**
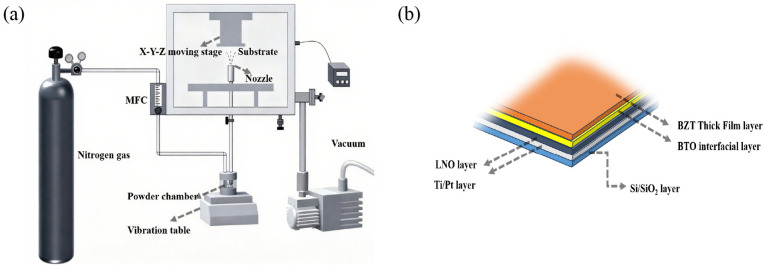
(**a**) Schematic illustration of the AD system. (**b**) Schematic of the BZT thick film heterostructure.

**Substrate Preparation:** The BZT thick films were deposited on BTO/LNO/Pt/Ti/SiO_2_/Si heterostructured substrates. As illustrated in Figure 1b, the architecture was built upon a silicon wafer (20 mm × 10 mm × 0.5 mm) possessing a thermally grown SiO_2_ layer. A Ti adhesion layer and a Pt bottom electrode were sequentially deposited via RF magnetron sputtering at 300 °C. A conductive LaNiO_3_ (LNO) layer and a thin BaTiO_3_ (BTO) buffer layer were subsequently deposited at an elevated temperature of 500 °C in an Ar/O_2_ (4:1) atmosphere.

**Aerosol Deposition Process:** The BZT thick films were deposited on the prepared substrates using a custom–built AD system, schematically depicted in Figure 1a. The prepared sub–micron BZT powder was loaded into an aerosol generator. Nitrogen (N_2_) gas was introduced at a flow rate of 12–15 L/min to fluidize and aerosolize the powder, assisted by mechanical vibration. Driven by the pressure differential between the aerosol generator and the deposition chamber (maintained at 220–300 Pa), the ceramic particles were accelerated through a Laval nozzle. These high–kinetic-energy particles impinged upon the substrate, fracturing and bonding to form a dense, nanocrystalline film via RTIC. Specific deposition parameters are detailed in Table 1.

**Figure A1:**
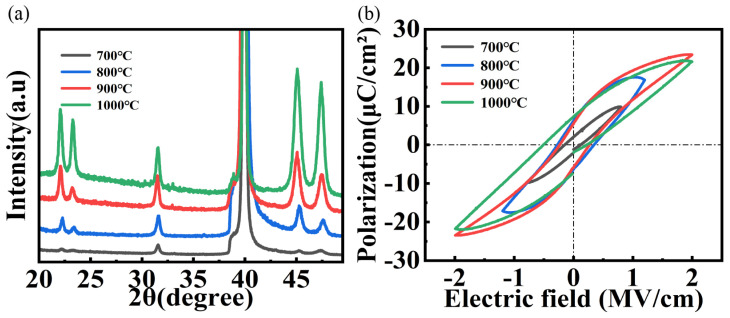
(**a**) Comparison of XRD patterns of BZT (*x* = 0.00) thick films at different annealing temperatures (**b**) *P*–*E* loops of BZT (*x* = 0.00) thick films at different annealing temperatures.

**Thermal Post-Treatment:** As–deposited films fabricated via AD are inherently characterized by substantial residual compressive stress and a highly refined nanocrystalline microstructure due to the impact consolidation mechanism. These features, while beneficial for density, can severely suppress the intrinsic ferroelectric and piezoelectric response. Accordingly, the as–deposited heterostructures (Figure 1b) were subjected to a stepped thermal annealing profile. The regimen consisted of sequential dwell times of 1 h at 500 °C, 700 °C, and 900 °C, followed by natural cooling to ambient temperature. This multi–stage heat treatment was indispensable for relaxing residual stresses and facilitating grain coarsening, thereby restoring the crystallinity and maximizing the electromechanical functionality of the films. The choice of 900 °C as the final annealing temperature was empirically determined to be optimal, as detailed in the Appendix A (Figure A1).

### 2.3. Structural and Morphological Characterization

The crystalline phase constitution of both the synthesized powders and the resultant thick films was determined via X–ray diffraction (XRD, Smart Lab 9 kW, Rigaku, Tokyo, Japan). To evaluate the surface topography and roughness evolution of the films, atomic force microscopy (AFM, AFM100 Plus, Hitachi, Tokyo, Japan) was utilized. Furthermore, scanning electron microscopy (SEM, FlexSEM 1000 II, Hitachi, Tokyo, Japan) was employed to conduct detailed microstructural examinations, specifically targeting the granular morphology of the powders and the cross–sectional morphology densification of the fabricated thick films.

### 2.4. Electrical and Piezoelectric Characterization

To facilitate electrical measurements, an array of circular Au top electrodes (*ϕ* = 200 μm) was patterned onto the BZT film surface via sputtering with a shadow mask (SBC–12, KYKY Technology, Beijing, China), thereby forming a parallel-plate metal–ferroelectric-metal (MFM) capacitor structure. Ferroelectric hysteresis loops and leakage current characteristics were acquired using a ferroelectric test system (Multiferroic, Radiant Technologies, El Segundo, CA, USA). Dielectric measurements, including capacitance–voltage (*C–V*) and frequency–dependent capacitance spectra (*C–f*), were performed utilizing a precision LCR meter (TH2838H, Tonghui, Changzhou, China).

The effective transverse piezoelectric coefficient, |*e*_31,_ *_f_*|, was determined via the cantilever deflection method [28]. The BZT/Si heterostructures with a Pt top electrode (~12 mm × 3 mm) were diced into rectangular shapes with dimensions of 20 mm × 3 mm × 0.5 mm. A unipolar sinusoidal voltage was applied to the top and bottom electrodes via a gold wire attached with silver paste. The resulting displacement at the free end of the cantilever was recorded using a Polytec laser Doppler vibrometer (LDV; OFV–505, Polytec Corp., Karlsruhe, Germany).

## 3. Results and Discussion

**Figure 2:**
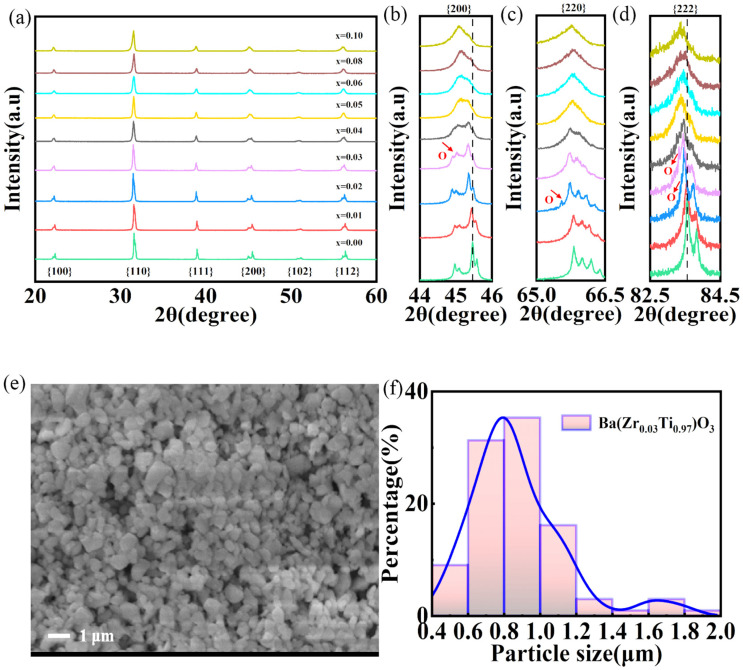
Structural and morphological characterization of the synthesized BZT powders. (**a**) XRD patterns of compositions with x ranging from 0 to 0.10. (**b**–**d**) Zoomed–in scans of the {200}, {220}, and {222} diffraction peaks, respectively. (**e**) Representative SEM micrograph and (**f**) particle size distribution histogram of the optimal Ba(Zr_0.03_Ti_0.97_)O_3_ powder.

The quality of the starting powder is paramount for both the AD process and the electromechanical properties of the final film. XRD (X–ray diffraction) analysis confirms the synthesis of phase–pure perovskite BZT powders across all compositions, with no detectable secondary phases (Figure 2a). A close inspection of the diffraction profiles reveals a monotonic shift in the peaks toward lower 2*θ* angles with increasing Zr content (Figure 2b–d). This trend indicates a systematic unit cell expansion, attributed to the successful substitution of larger Zr^4+^ cations for Ti^4+^ within the B-site sub-lattice. Crucially, the features of the diffraction profile around *x* = 0.03 provide direct evidence for the engineering of a critical phase state. Specifically, the characteristic broadening and asymmetric line shape of the {200} diffraction peak, coupled with the emergence of a new shoulder (Figure 2b), collectively signify the coexistence of tetragonal and orthorhombic phases. This engineered phase boundary near room temperature aligns with established phase diagrams [25] and serves as the fundamental origin of the enhanced electromechanical response explored in this work. Regarding morphological suitability, SEM analysis (Figure 2e) and particle size statistics (Figure 2f) reveal that the *x* = 0.03 powder consists of uniform, sub–micron particles with an average diameter of 0.87 µm ± 48 nm (Nano Measurer^TM^ software 1.2.5, Fudan University, Shanghai, China). This sub–micron geometry is essential for maintaining stable aerosolization and, consequently, achieving dense and high–quality thick films.

**Figure 3:**
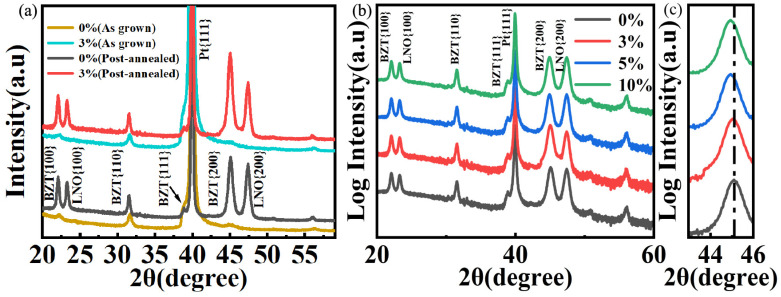
XRD analysis of BZT thick films. (**a**) 2θ scans of the as-grown and post-annealed films (linear intensity) (**b**) 2θ scans of representative compositions, confirming the formation of a pure perovskite phase. (**c**) Magnified view of the {200} reflection, highlighting the progressive peak shift, which is indicative of lattice expansion caused by Zr^4+^ incorporation.

The densification of BZT thick films via AD is fundamentally driven by the RTIC mechanism. During deposition, high–velocity ceramic particles impinge upon the substrate, where their kinetic energy is converted into bonding energy through a sequence of fracturing and plastic deformation. This violent impact induces severe comminution of the feedstock powder, effectively refining the micrometer-scale particles into a nanocrystalline matrix. Consequently, the as–deposited films exhibit broad, low–intensity diffraction patterns (Figure 3a), a hallmark of the reduced crystallite size and significant residual stress inherent to the AD process. Quantitative analysis using the Williamson–Hall method indicates an average grain size of approximately 22 ± 1 nm in the as-deposited state. Post–deposition annealing significantly enhances the crystallinity of the thick films. This thermal treatment facilitates grain growth, evidenced by the sharpening of diffraction peaks and an increase in the calculated crystallite size to a uniform ~78 ± 2 nm across all compositions (The relevant details are shown in the Appendix A Table A1). Crucially, the annealed films maintain a pristine single–phase perovskite structure without secondary phases, as corroborated by the X–ray diffraction patterns in Figure 3b.

Notably, in distinct contrast to the well-defined tetragonal splitting evident in the feedstock powders, the thick films exhibit merged, quasi-symmetric reflections characteristic of a macroscopic pseudo–cubic symmetry. This structural modulation is ascribed to the unique energetic conditions of the AD process; specifically, the nanocrystalline microstructure induced by the AD process, coupled with substantial residual stress, suppresses the effective c/a ratio and leads to the coalescence of diffraction peaks. A systematic migration of the {200} reflections toward lower diffraction angles is observed with increasing Zr content (Figure 3c). This trend corresponds to a monotonic lattice dilation—with the pseudocubic {200} expanding from 4.008 Å to 4.037 Å—thereby verifying the effective incorporation of larger Zr^4+^ ions into the Ti^4+^ lattice sites.

**Figure 4:**
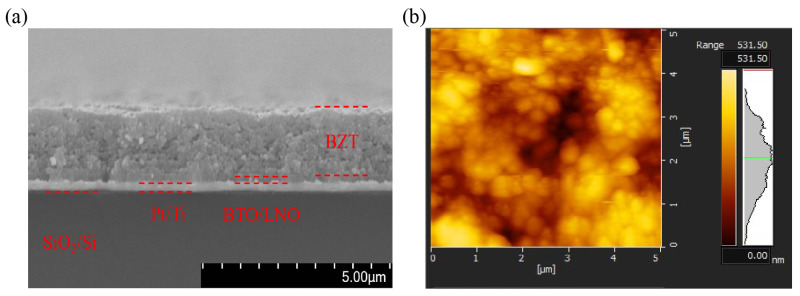
(**a**) Cross–sectional SEM and (**b**) AFM surface morphology (*x* = 0.03) of a post-annealed BZT thick film.

A key advantage of the aerosol deposition (AD) method is its ability to produce thick films with superior microstructural quality, which is confirmed by cross–sectional SEM and surface AFM analyses. As depicted in Figure 4a, the post–annealed thick film (~2 μm) possesses a remarkably dense and crack-free microstructure—a feature critical for ensuring device reliability and strong adhesion to the substrate. The high–magnification surface morphology, imaged by AFM (Figure 4b), further highlights a homogenous and tightly packed assembly of nanocrystalline grains. The average grain size was determined to be ~78 nm, which is in agreement with the XRD analysis, while the surface root–mean–square (RMS) roughness is measured as 30.0 nm. In comparison to the micrometer–scale grain size of the raw powder, this distinctive nanocrystalline morphology is a signature of the RTIC mechanism. During deposition, the massive kinetic energy of impinging particles is instantaneously converted into fracture energy and thermal activation, facilitating severe plastic deformation and cold–welding. The resulting pore–free, nanocrystalline matrix is critical, as it serves as the structural foundation for the high dielectric breakdown strength and enhanced piezoelectric displacement reported herein.

**Figure 5:**
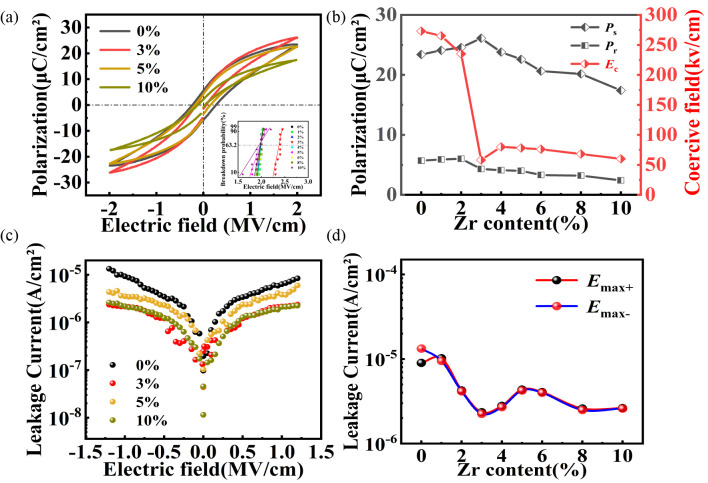
Ferroelectric properties of annealed BZT thick films: (**a**) *P–E* loops and (**b**) extracted *P*_s_, *P*_r_, and *E*_c_ (at 2 MV/cm). (**c**) *J–E* curves and (**d**) leakage current at ±1.2 MV/cm, plotted against Zr content. Inset: probability distribution of Weibull breakdown field strength.

The influence of the engineered phase boundary on the electrical properties is demonstrated by the ferroelectric behavior of the BZT films (Figure 5a,b). A clear performance optimum is identified at the critical composition of *x* = 0.03, which exhibits a remarkable combination of high maximum polarization (*P*_max_ = 31.3 μC/cm^2^ at 2.4 MV/cm) and a sharp minimum in the coercive field (*E*_c_ = 58 kV/cm). Although the characteristic peak splitting is merged in the thick film XRD patterns (Figure 3) due to grain refinement, the simultaneous enhancement in polarization and switching dynamics is consistent with the presence of a phase boundary. Based on the phase evolution observed in the precursor powders (Figure 2) and the dielectric anomalies, this performance enhancement is attributed to the underlying coexistence of tetragonal (T) and orthorhombic (O) phases. In the vicinity of this phase boundary, the free–energy profiles are significantly flattened, thereby reducing the energy barrier for both electric–field–induced phase transitions and non-180° domain wall motion, resulting in the observed “soft” ferroelectric response.

Conversely, as the Zr content exceeds this threshold (*x* > 0.03), the system compositionally shifts towards the O–R phase boundary. While the rhombohedral distortion is not distinctly resolved in the film XRD due to peak broadening, the retention of a low *E*_c_ and the evolution of electrical properties suggest the influence of the emerging rhombohedral phase. Although a low *E*_c_ is retained, the emergence of the rhombohedral phase—featured by multiple equivalent polarization axes—may lead to a reduction in the net macroscopic polarization under an applied electric field. Crucially, these ferroelectric enhancements are supported by the superior electrical integrity of the films. The relatively low *P*_r_ may be attributed to the substrate clamping effect or nanosized grains, rather than leakage current, as evidenced by the low current density in Figure 5c. The leakage current characteristics (Figure 5c,d) demonstrate excellent insulating behavior, with the optimal film exhibiting a minimized leakage current density of 2.35 × 10^−6^ A/cm^2^ at 1.2 MV/cm. This low leakage response is a direct testament of the dense, nanograined microstructure uniquely enabled by the AD process, ensuring that the measured properties are intrinsic to the film.

**Figure 6:**
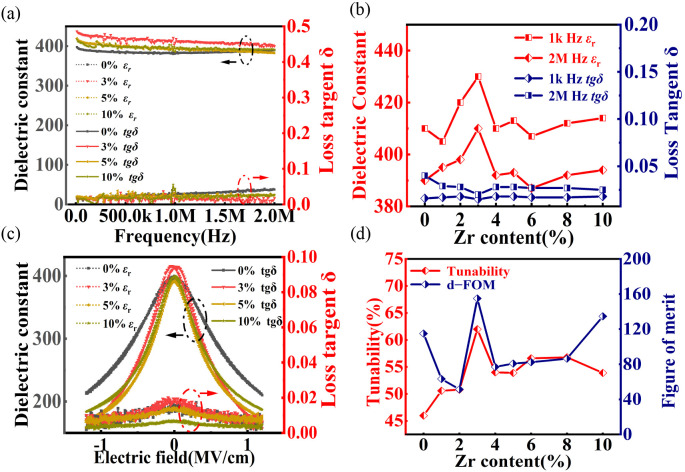
Dielectric properties of annealed BZT films. (**a**) Frequency dependence of relative permittivity (*ε_r_*) and dielectric loss (tan*δ*) measured from 20 Hz to 2 MHz. (**b**) Compositional evolution of *ε_r_* and tg*δ* at representative frequencies of 1 kHz and 2 MHz. (**c**) Variation in dielectric properties as a function of applied DC electric field. (**d**) Calculated dielectric tunability (*T*) and figure of merit (d–FOM) versus Zr content at a maximum field of 1.2 MV/cm.

Dielectric characterization provides further evidence for the enhanced electromechanical activity observed at the phase boundaries. The high quality of the AD-fabricated films is confirmed by their excellent dielectric stability, characterized by negligible frequency dispersion in both permittivity (*ε_r_*) and dielectric loss (tan*δ*) across a wide spectrum from 20 Hz to 2 MHz (Figure 6a). At a quasi-static frequency of 1 kHz (Figure 6b), the dielectric properties peak at the critical composition, with the *x* = 0.03 film exhibiting a maximum *ε*_r_ of ~430 coupled with a low tanδ of 0.015. Crucially, the DC bias-dependent measurements (Figure 6c) indicate the dynamic nature of the dielectric properties. According to the *tgδ*-*E* curves, the dielectric tunability (*T* = (*ε*_r(0)_ − *ε*_r(E)_)/*ε*_r(0)_, where *ε*_r(0)_ and *ε*_r(E)_ are the dielectric constants at zero and maximum DC bias electric field, respectively) and the dielectric figure of merit (d-FOM = *T*/*tgδ*, where *tgδ* is the average dielectric loss near the bias electric field) can be calculated. These two parameters are key indicators for characterizing the dielectric tunable properties of the material. The derived dielectric tunability (*T*) and figure of merit (FOM), plotted as a function of Zr content in Figure 6d, underscore the superior performance of the phase boundary composition. The film with *x* = 0.03 exhibits an exceptional dielectric tunability of 62.9% alongside a high figure of merit (FOM) of 155. Such elevated tunability is indicative of a “soft” ferroelectric state, which implies a more pronounced phase transition. Under external fields, this phase transition behavior facilitates polarization rotation, thereby contributing to the improved dielectric tunability. This result serves as a critical bridge between ferroelectric and piezoelectric properties, as high dielectric tunability is generally a precursor to a large piezoelectric response. It strongly suggests that the same mechanism—the flattened energy landscape—that optimizes the ferroelectric switching is also priming the material for superior piezoelectric performance.

To realize the superior electromechanical performance detailed above, two critical processing challenges inherent to the aerosol deposition method were systematically addressed. Primarily, to avoid the bottom electrode degradation induced by high-velocity particle bombardment, a highly oriented BaTiO_3_ buffer layer was integrated into the film architecture. This buffer acts as a mechanical shield, preserving the structural integrity of the film–electrode interface—a prerequisite for suppressing leakage currents and sustaining the exceptional breakdown fields (*E*_max_ ≈ 2400 kV/cm) demonstrated in Table 2. Complementing this interfacial engineering, a tailored multi-step annealing protocol was developed to overcome the limitations of the as–deposited state, specifically its significant residual stress and fine nanocrystalline morphology (∼21 nm). Dwells at 500 °C and 700 °C are essential for stress management rather than just crystallization. AD films possess immense residual compressive stress due to the impact consolidation mechanism. Direct heating to 900 °C typically induces thermal shock, leading to delamination or micro-cracking due to the thermal expansion mismatch between the ceramic film and the substrate. The stepped dwelling allows for gradual stress relaxation and defect annihilation, ensuring the integrity of the film–substrate interface.

This thermal treatment proved instrumental in relaxing internal stress while simultaneously driving significant secondary grain growth, yielding a dense, more perfect crystalline microstructure. The synergy between this robust, defect-minimized interface and the optimized bulk crystallinity underpins the enhanced ferroelectric and dielectric stability reported herein, ultimately unlocking the outstanding piezoelectric response that distinguishes this work.

The macroscopic electromechanical performance was assessed using millimeter-scale BZT thick film cantilevers. To quantify the piezoelectric response, the effective transverse piezoelectric coefficient, |*e*_31,_ *_f_*|, was extracted from the dynamic tip deflection data based on the cantilever beam theory [33]:

(1) $$e_{31,\;f}=-\frac{h_{s}^{2}Y_{s}}{3(1\;-{\;\nu }_{s})L^{2}V}\delta$$

In this expression, *δ* denotes the tip displacement measured under a unipolar sinusoidal driving voltage of amplitude *V*; while *L* is the active length of the cantilever from the clamping end to the laser point; where *h*_s_, *Y*_s_ (130 GPa), and *v*_s_ (0.278) correspond to the substrate thickness, Young’s modulus, and Poisson’s ratio of the Si substrate, respectively.

**Figure 7:**
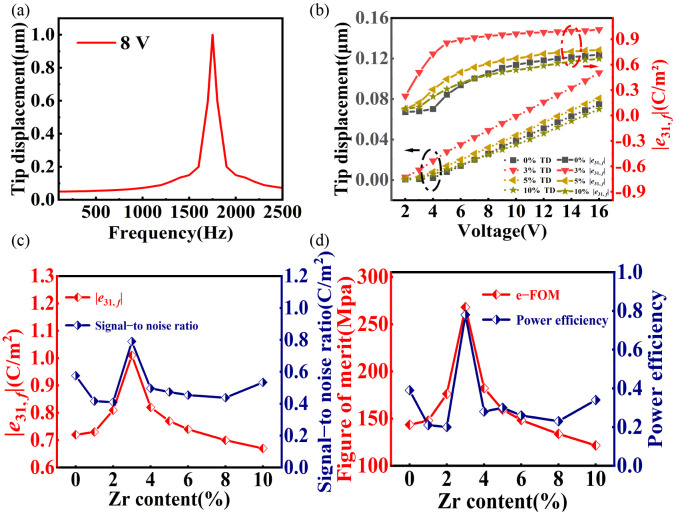
Piezoelectric characterization of annealed BZT thick film cantilevers. (**a**) Frequency dependence of the tip displacement. (**b**) Voltage dependence of the tip displacement and effective |*e*_31,_ *_f_*|. (**c**) |*e*_31,_ *_f_*| and SNR, and (**d**) e–FOM and power efficiency, as a function of Zr content.

The macroscopic transverse piezoelectric response serves as the direct confirmation of the phase boundary engineering strategy. To preclude resonant enhancement and ensure the measurement of intrinsic material properties, all tests were conducted at a driving frequency of 500 Hz, well below the cantilever’s resonance frequency of 1780 Hz (Figure 7a) As depicted in Figure 7b, the effective transverse piezoelectric coefficient, |*e*_31,_ *_f_*|, exhibits a pronounced dependence on Zr concentration. A distinct maximum is achieved at the critical composition of *x* = 0.03, which not only results in a rapid improvement in |*e*_31,_ *_f_*| under a much lower voltage (below 5 V) but also yields a superior |*e*_31,_ *_f_*| value of 1.01 C/m^2^ at 16 V. This performance represents a ∼50% enhancement relative to the undoped one and, notably, rivals the highest values reported for (001)–oriented BTO films fabricated via magnetron sputtering. The field-dependent evolution of |*e*_31,_ *_f_*| displays a characteristic sharp rise at sub-coercive fields followed by saturation. This phenomenon indicates that the extrinsic contributions—specifically, field-induced phase transitions that facilitate non-180° polarization rotations—are effectively activated and subsequently stabilize as the film approaches a fully poled state [33]. To assess the comprehensive device potential, the piezoelectric figure of merit (e–FOM = (*e*_31,_ *_f_*)^2^/(*ε*_r_*ε*_0_)), power efficiency (2(*e*_31,_ *_f_*)^2^(1 − *ν*_s_)/(*tanδε*_r_*ε*_0_*Y*_s_)), and signal-to-noise ratio (SNR = ∣*e*_31,_ *_f_*∣/(*ε*_r_*tanδ*)^1/2^) are key parameters that reflect the piezoelectric and overall electromechanical performances of the material, where *ε*_0_ is the permittivity of vacuum, and *ε*_r_ is measured at 500 Hz. As compellingly illustrated in Figure 7c,d, the optimization is holistic: the |*e*_31,_ *_f_*|, signal–to–noise ratio (SNR, 0.79 C/m^2^), piezoelectric FOM (268 MPa), and power efficiency (0.78) all exhibit a concomitant peak at *x* = 0.03. This synchronization underscores the pivotal role of the engineered phase boundary in simultaneously maximizing multiple functional parameters, thereby positioning aerosol-deposited BZT thick films as competitive candidates for next–generation, silicon-integrated lead–free piezoelectric applications.

## 4. Conclusions

This study establishes a robust fabrication route for dense, crack–free, and lead–free Ba(Zr*_x_*Ti_1−_*_x_*)O_3_ thick films on silicon by coupling compositional phase boundary engineering with the room temperature Aerosol Deposition process. Our investigation pinpointed *x* = 0.03 as the critical composition for stabilizing a polymorphic phase boundary (PPB), characterized by the coexistence of tetragonal and orthorhombic symmetries. This engineered multiphase structure effectively flattens the thermodynamic free-energy landscape, thereby facilitating easier phase transformation and enhancing polarization rotation. Consequently, the optimized BZT films demonstrate superior electrical performance, boasting a saturation polarization (*P*_max_) of 31.3 μC/cm^2^, a dielectric tunability of 62.9%, and a remarkable transverse piezoelectric coefficient, |*e*_31,_ *_f_*|, of 1.01 C/m^2^. Notably, these properties are commensurate with those of high–quality (001)–oriented sputtered BaTiO_3_ films, yet are realized with the distinct advantages of high deposition rates and cost-effectiveness inherent to the AD process. By reconciling high functional performance with direct–on–silicon integration, these findings position compositionally tuned, AD–deposited BZT thick films as a compelling material platform for next–generation, silicon–compatible piezoelectric MEMSs, particularly for high–performance actuators and energy harvesters.

## Figures and Tables

**Table 1:** Detailed parameters for the aerosol deposition process.

Parameter	Value
Deposition Pressure	220–300 Pa
Gas Flow Rate	12–15 L/min
Carrier Gas	N_2_
Nozzle-to-Substrate Distance	5 mm
Vibration Speed	500 rpm
Substrate Dimensions	20 mm × 10 mm

**Table 2:** Ferroelectric properties of AD-deposited BaTiO_3_–based thick films: a comparison. *P*_max_: maximum polarization (μC/cm^2^); *P*_r_: remnant polarization (μC/cm^2^); *E*_b_: breakdown electric field (kV/cm); *ε*_r_@1 kHz; YSZ: Yttrium-stabilized zirconia; SUS: Steel Use Stainless. kovar^®^ foils: a specially designed alloy substrate.

Ref. No	Substrate	Film	Annealing Temperature (°C)	*E*_b_ (kV/cm)	*P*_max_ (μC/cm^2^)	*P*_r_ (μC/cm^2^)	*ε*_r_	Loss *tgδ*
This work	Si	BZT(3/97)	900	2400	31.3	4.3	430	0.015
This work	Si	BTO	900	2000	23.4	5.7	410	0.016
[20]	304 SUS	BTO	750	500	5.5	2.5	N/A	N/A
[29]	SUS	BTO	1200	100	12.5	2.4	2200	0.02
[30]	SUS	BTO	1000	50	15	2.2	3070	N/A
[31]	YSZ	BTO	1100	100	15	4	3340	N/A
[32]	Free-standing	BTO	1100	65	24	5	2800	N/A
[21]	kovar^®^ foils	BTO	900	100	17.5	6.6	N/A	N/A

## Data Availability

The original contributions presented in this study are included in the article. Further inquiries can be directed to the corresponding authors.

## Appendix A

To determine the optimal post-deposition annealing temperature, BZT (*x* = 0.00) thick films were annealed at different final temperatures (700 °C, 800 °C, 900 °C, and 1000 °C) for 1 h. As shown in Figure A1a, the {110} peak becomes sharper and more intense with increasing annealing temperature, indicating enhanced crystallinity. The *P–E* loop measurements (Figure A1b) confirm this trend: the films annealed at 700 °C and 800 °C show small electrical breakdown strength with underdeveloped loops, whereas the 900 °C annealed film displays a better-saturated and robust ferroelectric hysteresis loop, indicative of superior electrical properties. However, further increasing the temperature to 1000 °C resulted in film cracking and delamination due to excessive grain growth and thermal stress, making it unsuitable for device fabrication. Therefore, 900 °C was confirmed as the optimal annealing temperature, providing the best balance between crystallinity, electrical performance, and film integrity. A comprehensive comparison (Table A1) also reveals that 900 °C annealing yields the optimal balance of large grain size, minimal residual stress, and lowest leakage, resulting in the maximized piezoelectric coefficient *e*_31,_ *_f_*.

**Table A1:** Grain size and lateral piezoelectric properties of BZT (3/97) thick films at different annealing temperatures.

Temperature (°C)	As Grown	700	800	900	1000
Average grain size (nm)	22 (1)	44 (1)	58 (2)	78 (2)	140 (3)
Strain (%)	−0.59 (0.02)	−0.2 (0.01)	−0.1 (0.01)	0.05 (0.01)	0.4 (0.02)
Leakage current (A/cm^2^)	2 × 10^−5^	8 × 10^−6^	4 × 10^−6^	2.35 × 10^−6^	8 × 10^−5^
*|e*_31,_ *_f_*| (C/m^2^)	N/A	0.05	0.6	1	0.8
